# Studies on Jackfruit–Okra Mucilage-Based Curcumin Mucoadhesive Tablet for Colon Targeted Delivery

**DOI:** 10.3389/fphar.2022.902207

**Published:** 2022-07-01

**Authors:** Pallavi Kurra, Kishore Narra, Raha Orfali, Srinivasa Babu Puttugunta, Shah Alam Khan, Dhanalekshmi Unnikrishnan Meenakshi, Arul Prakash Francis, Syed Mohammed Basheeruddin Asdaq, Mohd. Imran

**Affiliations:** ^1^ Vignan Pharmacy College, Gundur, India; ^2^ Department of Pharmaceutical Technology, BIT Campus, Anna University, Tiruchirappalli, India; ^3^ Department of Pharmacognosy, College of Pharmacy, King Saud University, Riyadh, Saudi Arabia; ^4^ College of Pharmacy, National University of Science and Technology, Muscat, Oman; ^5^ Centre of Molecular Medicine and Diagnostics (COMManD), Saveetha Dental College and Hospitals, Saveetha Institute of Medical and Technical Sciences, Chennai, India; ^6^ Department of Pharmacy Practice, College of Pharmacy, AlMaarefa University, Riyadh, Saudi Arabia; ^7^ Department of Pharmaceutical Chemistry, Faculty of Pharmacy, Northern Border University, Rafha, Saudi Arabia

**Keywords:** Curcumin, mucilage, jack fruit, okra gum, mucoadhesive

## Abstract

The present work investigates a blend of jack fruit mucilage (JFM) and okra mucilage (OKM) as promising mucoadhesive carriers for colon-specific delivery of a curcumin (CMN)-loaded mucoadhesive tablet (CMT) formulation. Formulation optimization was performed using central composite design (CCD) to further decipher the effect of varying proportions of the mucoadhesive carriers JFM and OKG on response factors such as drug release (% DR) and mucoadhesive strength (MA). The optimized formulation CMT (F14) demonstrated a favorable 54.35% *in vitro* release of CMN in 12 h with release kinetics resulting from a zero-order anomalous diffusion mechanism and MA of 34.1733 ± 1.26 g. Accelerated stability testing of CMT (F14) confirmed a shelf life of about 4.7 years. *In vivo* drug targeting studies performed using rabbit models in order to observe transit behavior (colon-specific delivery) of the dosage form were assessed by fluoroscopic images of the GI tract. Taking the results together, the results confirm that the combination of JFM and OKM could be exploited as an ideal mucoadhesive carrier for effective delivery of macromolecules to the colon.

## Introduction

Natural gums and mucilages are plant-derived biopolymers that have gained increased attention for pharmaceutical applications owing to their abundance, low cost, structural diversity, and excellent biodegradability. Natural mucilages have particularly been exploited by pharmaceutical industries for drug delivery as binders, bioadhesives, viscosity enhancers, suspending agents, thickeners, gelling agents, sustained-release polymers, etc., (Prajapati et al., 2013). These biological macromolecules with wide structural diversity can potentially replace synthetic excipients because of their superior hydrating ability, higher water-binding capacity, and swelling properties. These natural polymers also constitute potent targeting vehicles with enhanced GI residence time and thus could facilitate localized action of drugs present in the dosage form (Malviya et al., 2011).

Jack Fruit Mucilage (JFM) is a complex carbohydrate polysaccharide extracted from *Artocarpus heterophyllus Lam., (Moraceae)* fruit pulp. It comprises one or more monosaccharides and their derivatives. Several earlier studies have reported the use of JFM as an excipient in various mucoadhesive formulations such as microspheres, buccal tablets, controlled release tablets, etc., (Sabale et al., 2012). This is also a rich source of phytonutrients that are responsible for its wide spectrum of pharmacological activities, viz., antioxidant, anti-inflammatory, anti-helminthic, antidiabetic, anti-ulcerative, anti-cancer properties, etc., (Ranasinghe et al., 2019). The current study was an attempt to discover and unveil the underutilized potential applications of the mucilage obtained from the jackfruit waste processing in the development of site-specific drug delivery systems.

Okra Mucilage (OKM) obtained from the pods of *Abelmoschus esculentus (L.) Moench (Malvaceae)* is a randomly coiled pectic polysaccharide comprising multiple monomer units of rhamnose and galacturonic acid residues with disaccharide side chains. Apart from the antidiabetic activity shown by OKM polysaccharide rhamnogalacturonan, it has been reported to possess various medicinal applications such as anti-inflammatory, anti-irritant activity, serum albumin extender properties, etc., (Gemede et al., 2015). OKM has also been used as a binder/release retardant (Tavakoli et al., 2008), deflocculating agent/suspending agent (Ikoni, 2011), emulsifying agent (Alba et al., 2013), and mucoadhesive in various formulations such as buccal tablets, suspensions, gels (Sharma et al., 2013), sustained release tablets, mucoadhesive microspheres (Sinha et al., 2015), and other applications (Mohammadinejad et al., 2020).

We, therefore, contemplated that a combination of JFM and OKM biopolymers, because of their excellent mucoadhesive properties, could constitute an excellent bio-carrier for the development of a colon-targeted tablet formulation for controlled drug release.

Curcumin (CMN) is a well-established nutraceutical with varied clinical applications such as anticancer, cytoprotective, antioxidant, anti-inflammatory, and antidiabetic action (Sunagawa et al., 2015). However, CMN, classified as a biopharmaceutical classification system (BCS) class II drug, suffers from a stunted therapeutic index due to low aqueous solubility and bioavailability. The bioavailability of CMN has been improved by adopting several formulation techniques, especially the mucoadhesive formulation approach (Karki et al., 2017). The current study aimed to enhance the bioavailability of CMN and its delivery to the colon for potent action. A solid dispersion technique with a known hydrophilic polymer was used for enhancing the bioavailability of CMN, whereas the anticancer and anti-ulcer potential of CMN was enhanced by developing localized drug delivery for colorectal cancer and colonic ulcers. The hydrophilic polymer, PVP K30, belonging to the family of PVP, is an amorphous high-molecular polymer with a high melting point and good aqueous solubility. After oral administration, PVP is not absorbed by the gastrointestinal tract and hence it is considered nontoxic. It is a commonly used carrier in solvent dispersion formulation protocols. PVK K30 has been commonly used as a hydrophilic carrier for enhancement of the compromised solubility of curcumin, which is an existing and traditional method for solubility enhancement of BCS class II drugs (Kurakula and Rao, 2020).

It was anticipated that the CMN colon-targeted drug delivery system might serve as an effective dosage form with specific drug targeting, reduced dose, decreased systemic side effects, and enhanced drug efficacy. The existing colon-targeted formulations such as guar gum–based tablets, microspheres, and nanoparticles have limitations such as unintentional disintegration, less capability to bind to the rough surface of the intestinal mucosa, compromised systemic bioavailability, etc., (Sareen et al., 2014).

In the current study, the colon-targeted drug delivery system formulated in the form of a CMN mucoadhesive tablet (CMT) containing a combination of JFM and OKM with sustained, site-specific delivery was developed. Optimization of the best CMT was performed using response surface methodology. The optimized CMT serves as an effective vehicle for drug targeting and thereby provides a better bioavailable, sustained-release colon-targeted drug delivery system.

## Materials and Methods

### Materials

Jack Fruit mucilage (JFM) and Okra mucilage (OKM) were extracted from the raw materials produced from the local market in Tiruchirappalli, Tamil Nadu, India. Curcumin (CMN) was obtained as a gift sample from Laila Nutraceuticals Pvt. Ltd., India. PVP K 30 was purchased from Sigma-Aldrich. All the other ingredients used were of analytical grade.

### Preparation of CMN Solid Dispersions

The CMN solid dispersions (CSDs) were prepared by the solvent evaporation technique using the hydrophilic carrier PVP K30. CMN and polymer in a ratio of 1:3 were dissolved in acetone under sonication. The residual solvent was evaporated by vacuum evaporation at 50°C in an electric water bath. The dried binary mixture was sieved (#66) to ensure uniform particle size distribution and was stored for further evaluation.

### Morphological Study of CSDs

The surface characteristics of pure CMN and CSDs were analyzed using a KYKY EM-3200 scanning electron microscope (KYKY, Beijing, China). The images were visualized at a magnification range of ×350 to ×1000. The difference in the structural features was evaluated.

### Estimation of Wetting Efficiency of CSDs by the Washburn Method

**FIGURE 1:**
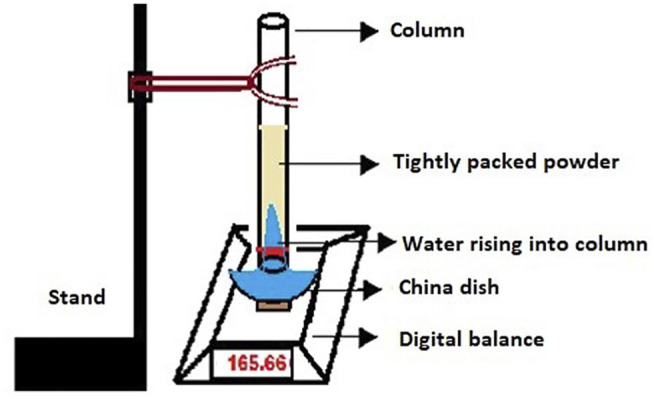
Illustration of the setup used for the estimation of the Washburn slope.

The arrangement of components to approximate wetting efficiency using the Washburn method (Kiesvaara and Yliruusi, 1993) is shown in Figure 1. The technique presumes the construction of a powder column which accommodates compacted powder material intended for testing. A digital weighing balance whose D = 0.1 mg is set underneath the column. Any wetting liquid functioning as water is taken in a china dish, placed on the weighing balance, and the total weight is noted down as the initial weight. The column is lowered until it touches the surface of the water. Because of the wetting and capillary action, water advances into the column from the china dish, resulting in a decrease in weight, displayed on the digital balance (Mettler Toledo, Switzerland). The alteration in the weight every 5 s is recorded. To have identical results, the packing of all powders is performed by tapping the columns. The mass of water raised every 5 s is computed with the help of Eq. 1:

Mass of water raised for every 5 seconds=Initial weight of water−Final weight of water. (1)

According to Washburn’s method, a graph was plotted by considering time on the *X*‐axis and mass on the *Y*‐axis, whose slope is η /C. ρ^2^. γ. cos θ. The greater the slope of the graph, the greater the wetting efficiency will be (Prestidge and Tsatouhas, 2000).

### Solubility Analysis

The solubility of pure CMN was estimated with water as a medium (Kurra, 2018). CMN alone and a 1:3 ratio of CMN–PVP solid dispersion were added to water and stirred using a magnetic stirrer at 300 rpm for 48 h at 37 ± 0.5°C. The prepared samples were filtered using 0.10-µm pore size Millex-VV PDFV filters with the aid of a one-step vacuum pump. All samples were suitably diluted with the respective medium and analyzed for CMN content spectrophotometrically at 421 nm using an Elico SL 210 double beam UV–visible spectrophotometer (Elico SL-210, Hyderabad, India).

### Preparation of CMT

In order to achieve the objective of CMN colon targeting and localized drug delivery, a measured amount of CSD (1:3 ratio) was mixed homogeneously with a mucoadhesive blend containing JFM and OKM (Souza Rocha et al., 2021). Based on the primary evaluations performed for a combination of JFM and OKM, (75 and 125 mg) and (150 and 250 mg) were fixed as low value and high value for both the mucoadhesive polymers, that is, JFM and OKM, respectively. All the ingredients as mentioned in Table 1 were weighed accurately, pulverized, and blended uniformly. The compression was performed using a 10 station rotary tablet compression machine. An amount of 550 mg was fixed as the total tablet weight.

**TABLE 1:** Composition of various CMN mucoadhesive tablet formulations.

Formulation code	CSD (mg)	JFM (mg)	OKM (mg)	Dicalcium phosphate (mg)	Magnesium stearate (mg)	Talc (mg)
M1	115	200	64.645	150.856	9.75	9.75
M2	115	200	100	115.5	9.75	9.75
M3	115	250	125	40.5	9.75	9.75
M4	115	200	135.35	80.145	9.75	9.75
M5	115	200	100	115.5	9.75	9.75
M6	115	200	100	115.5	9.75	9.75
M7	115	150	125	140.5	9.75	9.75
M8	115	150	75	190.5	9.75	9.75
M9	115	270.71	100	44.789	9.75	9.75
M10	115	129.29	100	186.211	9.75	9.75
M11	115	250	75	90.5	9.75	9.75
M12	115	200	100	115.5	9.75	9.75
M13	115	200	100	115.5	9.75	9.75

### Optimization of CMT

Statistical optimization was executed using Design-Expert^®^ Version 11.0 software (Stat-EaseInc., United States). An optimization technique using central composite design (CCD) was utilized to study the effect of quantities or proportions of natural polymers, that is, JFM/Factor A and okra mucilage OKM/Factor B on response factors such as cumulative drug release (% CDR) and mucoadhesive strength [MA in gram (g)].

### Characterization

Compressed mucoadhesive tablets were evaluated for various tableting properties such as hardness using a Monsanto hardness tester, thickness using vernier calipers, weight variation, content uniformity, and friability using a Roche friabilator. All the tests were performed according to the procedures mentioned in the USP monograph.

### FT-IR

Drug excipient compatibility studies were performed by Fourier transform infrared (FT-IR) spectrum analysis using the FT-IR Bruker 10048657 Alpha T instrument (Billerica, Massachusetts) over the range of 4,000 to 400 cm^−1^. CMN along with the selected excipients was analyzed individually and in combination with the polymers used in the formulation (Paradkar et al., 2004).

### Differential Scanning Calorimetry

DSC of pure CMN and the optimized mucoadhesive formulation (M-14) was performed using DSC 2920 Differential Scanning Calorimeter, United States, and data were analyzed by Universal Version 4.5A. Each sample weighing 5–10 mg was placed in sealed aluminum pans. Scanning of samples was performed at 40–400°C at a rate of 20°C per minute.

### Swelling Study

The swelling index, which is an indication for hydration of the mucoadhesive tablets, was studied in a simulated GI fluid medium at 1.2 pH (gastric pH) and 7.4 pH (colonic). The degree of swelling was measured by measuring the variation in the weight of the mucoadhesive tablets. All the pre-weighed mucoadhesive formulations (M1–M13) were placed into petri plates containing 50 ml of the test medium. The tablets were removed after designated time intervals, blotted with filter paper to remove excess medium, and subjected to reweighing. The swelling index was calculated using Eq. 2.

$$Swelling\;index\;\left(\%\right)\;=\frac{w_{2}-w_{1}}{w_{1}},$$ (2)

where W1 and W2 are the weights of mucoadhesive tablets before and after swelling, respectively.

### *In Vitro* Drug Release Study

*In vitro* drug release studies for all the proposed trial mucoadhesive formulations (M1–M13) were performed using 708-DS Dissolution Apparatus (from Agilent Technologies, United States) in various physiological pH conditions resembling various regions of GIT. The stirring speed was maintained at 75 rpm. A bowl temperature of 37 ± 0.5°C and a bath temperature of 37.5 ± 0.5°C were maintained throughout the study with a dissolution fluid volume of 900 ml. The dissolution study was performed for a total duration of 12 h. For the first 2 h, the dissolution was performed in 1.2 pH acidic media, followed by pH 7.4 simulated medium (colonic pH) for the rest of the duration. A 5-ml sample was withdrawn after each time interval, and perfect sink conditions were maintained. The samples were estimated for drug content spectrophotometrically using Elico SL 210 double-beam UV–visible spectrophotometer at λ_max_ 419 nm (Elico SL-210, Hyderabad, India) (Kurra, 2018). Cumulative % drug release (% CDR) was calculated and was considered the response factor for the optimization of the best mucoadhesive formulation.

### Mucoadhesive Strength

Mucoadhesive (MA) strength of all the trial mucoadhesive formulations (M1–M13) was evaluated as MA in gram using a modified digital balance apparatus as per the previously reported method (Kurra, 2018). The weight in grams required to detach the mucoadhesive formulation from the porcine stomach mucosa was estimated and was considered the response factor for the optimization of the best mucoadhesive formulation.

### *Ex Vivo* Dissolution Studies

To recognize the sensitivity of natural mucilages JFM and OKM to colonic microflora, an *ex vivo* dissolution study was performed using 2% rat cecal contents for the optimized formulation M14. The study was executed as per the details mentioned in the experimental protocol approved by the Institutional Ethics Committee (001/VPC/IAEC/RESEARCH-2018). The detailed methodology has been given in the Supplementary Information.

### Kinetic Modeling and Release Date Comparison

The dissolution data of the optimized formulation (M-14) in pH conditions (pH 1.2 and pH 7.4) were incorporated into various mathematical models such as zero-order, first-order, Higuchi, and Korsmeyer-Peppas models to establish the kinetics of drug release.

The similarity factor specified by Scale-up and Post Approval Changes (SUPAC) guidelines was used as a standard to compare the dissolution data with and without colonic content. Dissolution data without colonic contents were considered to be the reference and colonic contents were considered a test. The similarity between dissolution profiles is believed to exist if the f_2_ values lie between 50 and 100. The *f*_*2*_ value can be calculated using Eq. 3.

$$f_{2}=50\log\left[{\left\{1+\frac{1}{n}\sum_{t=1}^{n}{\left(R_{t}-\;T_{t}\right)}^{2}\right\}}^{-0.5}X\;100\right],$$ (3)

where *n* = dissolution time and *R*_*t*_ and *T*_*t*_ are the reference and test dissolution values at time t, respectively.

### Hemoaffinity Studies

The study was performed according to the previously reported method (Hu et al., 2015). Briefly, freshly collected rat blood was subjected to centrifugation at 2,000 rpm for 10 min to separate plasma from the blood cells (RBCs). The plasma obtained as the supernatant liquid was discarded, and the pellet consisting of RBC concentrate was suspended in a 7.4 pH phosphate-buffer solution (PBS). The resultant solution was further centrifuged at 2,000 rpm for 10 min. The formed supernatant was discarded, and the same step was repeated thrice until the entire buffy layer was removed. The concentrated RBCs obtained were made into a suspension by using PBS at a concentration of 2% v/v for further study. To 900 µl of the RBC suspension, 100 µl of various test compound solutions [JFM (T1), OKM (T2), JFM and OKM (T3), CMN (T4), and M14 formulation (T5)] with a concentration of 10 µM was added. RBC suspension treated with 100 µl double-distilled water acted as a positive control, whereas RBC suspension treated with 100 µl of 7.4 pH PBS acted as a negative control. The test samples were placed in a water bath for 1 h at 37°C. After 1 h, the samples were centrifuged at 2,500 rpm for 10 min. The absorbance of the supernatant solution was recorded at 541 nm using an Elico SL 210 double beam UV–visible spectrophotometer (Elico SL-210, Hyderabad, India).

### Accelerated Stability Testing

Methodology details are given in the Supplementary Material.

### *In Vivo* Targeting Study

To support the *in vitro* studies and mimic the human *in vivo* conditions, the optimized formulation was tested in an animal model and the transit behavior (colon-specific delivery) of the dosage form was observed. The current animal study was performed as per the procedure mentioned in the experimental protocol cleared by the Institutional Ethics Committee (001/VPC/IAEC/RESEARCH-2018).

New Zealand white rabbits (3–3.5 kg) were selected as the animal models to simulate human colonic environment physiology (Singh et al., 2020, Singh et al., 2021). The rabbits were starved overnight with free access to water. The optimized mucoadhesive formulation M14 was reformulated with barium sulfate in the mucoadhesive tablet, which serves as a radio-opaque material. The prepared tablets were administered to the rabbits orally through gastric intubation. X-ray or fluoroscopic imaging using the L and T Vision Pro 100 (C-arm) digital X-ray machine (Allengers Mars 15–80 fixed X-ray) was used to monitor the movement of the dosage form in the GI tract. Fluoroscopic images of the rabbit GI tract were taken at predefined intervals of 0, 1, 4, 6, and 12 h to track the staging of the administered formulation.

## Results and Discussions

For colon targeting, CSD was mixed with a mucoadhesive blend containing varying ratios of JFM and OKM, diluent, glidant, and lubricant. Except for the ratio of the mucoadhesive blend, all other parameters were maintained constant as shown in Table 1 throughout the study. The homogenous blend was compacted by the direct compression technique to obtain the tablets.

### Morphological Study of CSD

**FIGURE 2:**
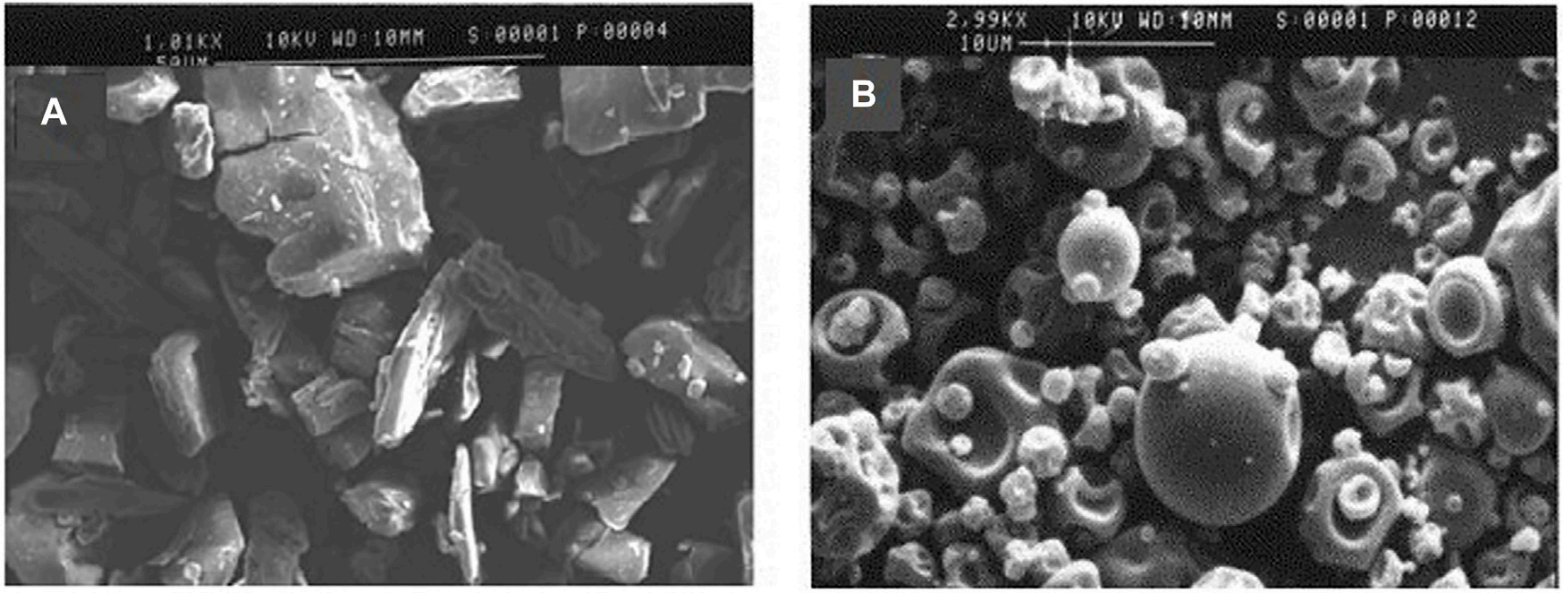
SEM images of **(A)** CMN and **(B)** CSDs prepared with PVP K 30 (1:3 ratio).

Morphological investigation of CMN (Figure 2A) under a SEM revealed flat, broken, irregular, and edged surfaces, while CSD (Figure 2B) exhibited regular, spherical-shaped particles, which confirms encapsulation of the drug in the carrier matrix. The adherence and aggregation of the CMN and PVP K 30 were also observed to change after the formation of solid dispersion owing to the conversion of a crystalline form of CMN into an amorphous form that has a higher surface area (Paradkar et al., 2004).

### Wetting Efficiency by the Washburn Method

The wetting efficiency of powder materials is related to the wicking flow of liquid in a capillary tube containing a packed column of powder (Figure 1). It is estimated from the slope of a curve plotted between Mass^2^ (mass of water raised into the column) versus time in seconds, where the sample with more slope is considered to have higher wetting efficiency.

**FIGURE 3:**
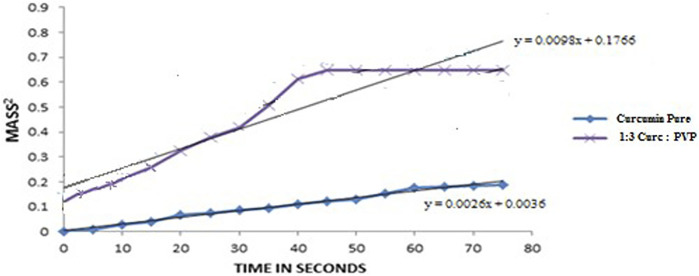
Washburn graph displaying the slopes of CMN pure drug and 1:3 ratio CSDs prepared with PVP K30.

The Washburn graph of CMN pure drug and 1:3 ratio CSD prepared with PVP K30 has been shown in Figure 3 and the Washburn slopes of pure CMN and CSD were found to be 0.0026 and 0.0098, respectively. It was observed that the wetting ability of CMN was increased (3.8 times) in the 1:3 ratio solid dispersion made with PVP K30 compared to that of pure CMN. The enhanced wetting may be attributed to changes in physical properties of CMN molecules, that is, conversion of crystalline form to amorphous form and formation of a soluble complex in the presence of PVP K30 (Xu et al., 2008).

### Solubility Studies

Solubility is a concomitant measure of dissolution and bioavailability. The solubility of pure CMN and CSD prepared with PVP K30 was found to be 25 ± 1.2 μg/ml and 300 ± 15 μg/ml, respectively. From the study, it is evident that a significant 12-fold increase in the solubility of CMN was observed with the addition of a hydrophilic carrier (PVP K30) that could be attributed to the association of CMN with the hydrophilic functional groups present in the carrier [18]. The enhanced solubility can also be ascribed to the formation of the most soluble complex with upgraded wettability as per Washburn results, increased hydrogen bonding capacity of CMN, and transformation of CMN into amorphous form by the solvent evaporation technique (Li et al., 2013).

### Outcomes From CCD

The right combination of process variables can yield a consistent product with the desired quality. In the current work, the mucoadhesive tablets of CMN were optimized in accordance with the statistical design CCD for reliable measurement of response variables. Based on the experimental results, suitable polynomial equations were developed, after which, statistical tests with the best fit model were selected, and thus the independent variables producing optimum response are reported.

For CCD, 13 trial formulations as shown in Table 2 were assigned by Design-Expert Software Version 11 (Stat-Ease Inc., United States). The two independent variables considered are the amount of JFM (Factor A) and the amount of OKM (Factor B) at two levels; low (−1) and high (+1). The response factors selected were % CDR and MA in gram. Table 2 gives the summary of the results obtained for all the CMN mucoadhesive tablets (M1–M13).

**TABLE 2:** Response variables of CMN mucoadhesive tablet.

Formulation code	JFM (mg) factor A	OKM (mg) factor B	% CDR	MA (gm)
M1	200	64.6447	63 ± 1.17	27 ± 0.17
M2	200	100	76 ± 1.38	23 ± 0.28
M3	250	125	43 ± 0.20	33 ± 0.94
M4	200	135.355	78 ± 1.18	23 ± 0.29
M5	200	100	78 ± 1.13	28 ± 1.76
M6	200	100	73 ± 1.17	20 ± 1.45
M7	150	125	88 ± 1.23	26 ± 1.34
M8	150	75	93 ± 1.82	33 ± 1.64
M9	270.711	100	49 ± 0.19	37 ± 0.58
M10	129.289	100	97 ± 1.50	27 ± 0.71
M11	250	75	68 ± 0.39	29 ± 0.68
M12	200	100	77 ± 0.098	39 ± 1.51
M13	200	100	74 ± 0.082	19 ± 1.76
M14	250.95	124.96	54.35 ± 2.13	34.17

The results of ANOVA for % CDR (quadratic model) and MA (linear model) represented in Supplementary Table S1 (Supplementary data) indicate that the model is significant for both the response parameters selected.

The model F value of 88.30 for response factor % CDR at 12 h and the model F-value of 136.15 for response factor MA imply the model is significant. As per literature, there is only a 0.01% chance for this larger F value to be CMN due to noise. *p*-values less than 0.05 indicate that the model terms are significant. In the CMN rent case, for % CDR response, A, B, A^2^, and B^2^ were significant model terms, whereas for response factor, MA, in g A and B were significant model terms. Any values greater than 0.1000 indicated in the model terms are not significant. Since there exists only one insignificant model term AB for the response % CDR and no insignificant terms for the response factor MA, model reduction is not required.

Lack of fit F-value of 0.48 and 1.40 for the response factors % CDR and MA, respectively, implies the lack of fit is found to be nonsignificant relative to the pure error indicating the positive fit statistics. The nonsignificant lack of fit also confirms that the model is fit for the response parameters used.

Fit statistics represent a correlation coefficient (*R* ^2^) value of 0.9844 and a coefficient of variance % of 3.09 for response factor % CDR and a correlation coefficient (*R* ^2^) value of 0.9646 and a coefficient of variance % of 4.53 for response factor MA in g. The predicted R^2^ of 0.9528 and 0.9646 for response factors % CDR and MA is in reasonable agreement with the adjusted R^2^ of 0.9732 and 0.9575, respectively; that is, the difference is less than 0.2. Adeq model precision measures the signal-to-noise ratio. A ratio greater than 4 is desirable. The obtained ratio of 30.573 and 32.084 for % CDR and MA, respectively, indicates an adequate signal and suggests that this model can be used to navigate the design space.

The polynomial Eqs 4, 5, relating to response factor % CDR, and MA, after model simplification, that is, eliminating nonsignificant terms in the model equation, are as follows:

$$\%\;CDR\;=\;+67.40\;-\;13.17*A\;-\;6.76*B\;+\;1.75\;AB\;+\;4.55\;A^{2}\;-\;2.70\;B^{2.}$$ (4)

MA = +28 + 6.76∗A + 3.02∗B. (5)

The final Eq. 6 in terms of actual factors can be used to make predictions about the response for given levels of each factor.

$$\%\;CDR\;=\;+67.40\;-\;13.17*JFM\;-\;6.76*OKM\;+\;1.75\;JFM\;OKM\;+\;4.55\;JFM^{2}\;-\;2.70\;OKM^{2}\;MA\;=\;+28\;+\;6.76*JFM\;+\;3.02*OKM.$$ (6)

**FIGURE 4:**
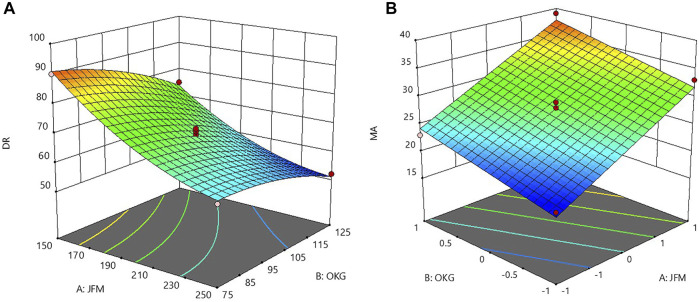
3D response surface plots showing the combined effect of JFM-OKM on **(A)** % CDR at 12 h and **(B)** MA.

The influence of main factors, namely, the weight of JFM and the weight of OKM on the responses of % CDR and MA was further explored by using the response surface methodology. Three-dimensional response surface plots as shown in Figures 4A,B indicate the combined effects of JFM and OKM on % CDR and MA in g. The 3D response surface plots correlating to R12h indicated decreased R12h values with an increase in independent variables (amount of JFM and OKM in CMT). This behavior may be due to the increased intermolecular crosslinking and network formation of JFM and OKM with an increase in wetting time (El-Shenawy et al., 2017).

The influence of polymer A: JFM was found to be more than that of polymer B: OKM. The 3D plot correlating to MA in g indicated increased mucoadhesive strength with an increase in the amount of JFM and OKM in the CMT. This might be due to the increased mucin binding sites obtained with increased mucoadhesive concentration (Xing et al., 2017).

Based on the graphical optimization and checkpoint formulation compositions, validation of the experimental design and polynomial equation optimization of the formulation were carried out. The optimized mucoadhesive formulation M14 containing JFM 250.95 mg and OKM 124.96 mg was evaluated for hardness, thickness, weight variation, friability, content uniformity, % CDR, and MA. The results obtained for weight variation, friability, content uniformity, % CDR, and MA were within the prescribed USP limits. The predicted % CDR values obtained from the Design expert software and the experimental values were concomitantly compared with experimental responses. The optimized formulation (M-14) showed experimental % CDR 12 h (54.35 ± 2.13%) and MA (34.1733 ± 1.26 g), whereas the predicted drug release value % CDR 12 h was found to be 51.07%, and the predicted MA was found to be 37.773 g. The minimal deviation between the two values indicates that the mathematical model is well-fitted and is significant. The result also signifies that the generated model is valid and robust.

### Evaluation of CMT

Compressed mucoadhesive tablets (M1–M13) were evaluated for various tableting properties such as hardness, thickness, weight variation, content uniformity, friability, disintegration, and dissolution. The results shown in Table 3 indicated that all the tablets passed the weight variation test and the friability test. The weight variation was found to be <5%, and the percent loss in the friability test was found to be <1% which is acceptable according to USP. Hardness was fixed between 3–5 kg m^−2^. Content uniformity was found to be between 99.19 and 99.49 ± 1.43 mg, which is within the limits prescribed by USP.

**TABLE 3:** Post-compression properties of mucoadhesive tablets.

Formulation	Weight variation (mg)	Friability (%)	Content uniformity	Swelling index	
				pH 1.2	pH 7.4
M1	1.01 ± 0.01	0.034 ± 0.001	99.18 ± 0.12	2.23 ± 0.1	18.3 ± 0.8
M2	1.18 ± 0.02	0.043 ± 0.001	99.13 ± 0.13	3.15 ± 0.1	25.6 ± 0.1
M3	1.09 ± 0.03	0.054 ± 0.001	99.23 ± 0.15	3.62 ± 0.1	34.3 ± 0.4
M4	1.03 ± 0.02	0.051 ± 0.001	99.19 ± 0.13	2.99 ± 0.1	35.6 ± 0.3
M5	1.03 ± 0.03	0.012 ± 0.001	99.13 ± 0.26	3.18 ± 0.1	26.3 ± 0.7
M6	1.04 ± 0.01	0.065 ± 0.001	99.26 ± 0.17	3.72 ± 0.1	25.3 ± 0.3
M7	1.11 ± 0.02	0.055 ± 0.001	99.32 ± 0.14	3.66 ± 0.1	19.5 ± 0.7
M8	1.12 ± 0.01	0.072 ± 0.001	99.49 ± 0.22	3.82 ± 0.1	17.8 ± 0.2
M9	1.07 ± 0.03	0.071 ± 0.001	99.37 ± 0.19	3.69 ± 0.1	29.2 ± 0.3
M10	1.04 ± 0.05	0.063 ± 0.001	99.26 ± 0.14	3.29 ± 0.1	27.6 ± 0.4
M11	1.03 ± 0.02	0.070 ± 0.001	99.29 ± 0.16	3.88 ± 0.1	27.5 ± 0.5
M12	1.09 ± 0.01	0.024 ± 0.001	99.16 ± 0.17	3.89 ± 0.1	26.2 ± 0.2
M13	1.10 ± 0.03	0.032 ± 0.001	99.43 ± 0.13	3.77 ± 0.1	25.2 ± 0.3

*data are presented as mean ± SD of triplicate test values.

### FT-IR Studies

The FT-IR spectrum of CMN showed characteristic deformation vibrations of both the benzene rings with peaks observed at 700–500 cm^−1^ and the out-of-plane vibrations accredited to the peak observed at 607 cm^−1^. The peak visible at 1031 cm^−1^ can be assigned to C-O stretching associated with C-C stretching vibrations. The band present at 1,124 cm^−1^ represents C-O-C vibrations. The C=C-H of interring chain can be accredited as a peak at 1,207 cm^−1^. The peak at the 1,167 cm^−1^ band and shoulder visible at 1,270/1,238 cm^−1^ can be attributed to the in-plane deformation vibration of C-C-H of phenyl rings and skeletal in-plane deformations. The band observed at 1,625 cm^−1^ can be ascribed to the stretching of C=C of the benzene ring. Low-intensity bands appearing in the region 2,700–3,000 cm^−1^ can be accredited to the presence of multiple C-H bond stretching and methyl group motions, and the broad peak at 3,380 cm^−1^ indicates the presence of O-H bond in the CMN molecule (Sampath Udeni Gunathilake et al., 2018).

All the characteristic peaks of CMN (pure drug) were obtained in the IR spectra of optimized mucoadhesive formulation M14, indicating no significant interaction between CMN and the selected excipients of the formulation M14, which also ensures that the drug remained intact in the mucoadhesive tablet.

### DSC Study

**FIGURE 5:**
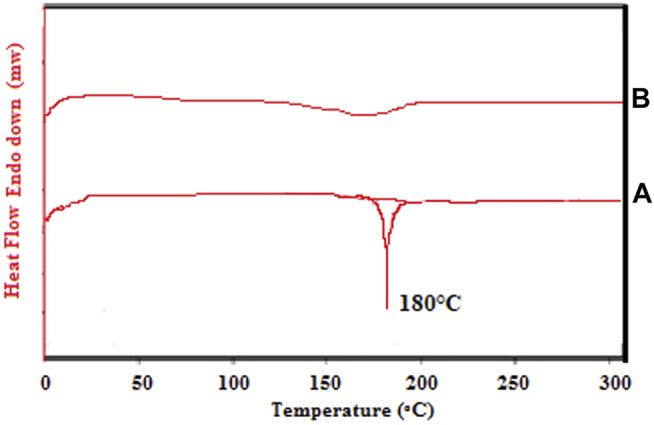
DSC thermograms [A-pure CMN and B-Optimized CMT (M14)].

The DSC is regarded as the most useful technique for analyzing the thermal behavior of solid dispersions. It also indicates changes in the drug’s behavior in the presence of excipients, aiding in the identification of incompatibility. The DSC thermograms of CMN are displayed in Figure 5. The data curve A manifested an endothermic peak at 180°C, which correlates with the melting point of the pure CMN. The DSC thermogram of optimized formulation M14 as shown in the data curve B manifested a drastic decrease in the intensity of the peak, indicating the conversion of a crystalline form of CMN to an amorphous form (Mendonça et al., 2015). This conversion can be attributed to the formed solid dispersion of CMN and PVP K30 by an evaporative precipitation technique.

### Swelling Index

The swelling index test was performed for all the mucoadhesive tablet formulations in altered pH conditions of the media at 1.2 and 7.4. The data presented in Table 3 show that all the formulations exhibited pH-responsive swelling that could be attributed to the presence of polysaccharides in JFM and OKM with negatively charged carboxyl groups. Significantly less swelling was observed in the pH 1.2 acidic media which can be due to the absence of any net charge on the surface of a tablet owing to the undissociated carboxyl groups. However, significantly higher swelling was observed in all the mucoadhesive formulations when exposed to pH 7.4 alkaline media owing to the negatively charged carboxylic acid ions and internal repulsions between polymer chains (Agarwal et al., 2015; Soleimani et al., 2021). The plausible reason behind the variable swelling behavior with variation in pH might be attributed to the higher hydrogen bonding capacity of mucoadhesive tablets in alkaline pH than in acidic pH medium. Furthermore, it was noted that the extent of swelling was increased with an increase in the concentration of mucilages present in the formulations (Emeje et al., 2011). The influence of JFM concentration on the swelling index was found to be higher than that of OKM concentration. The optimized M14 formulation was found to have a swelling index of 21.9 ± 0.2, making it optimal for a sustained drug release pattern.

### *In Vitro* Drug Release and Kinetic Analysis of Release Data

**FIGURE 6:**
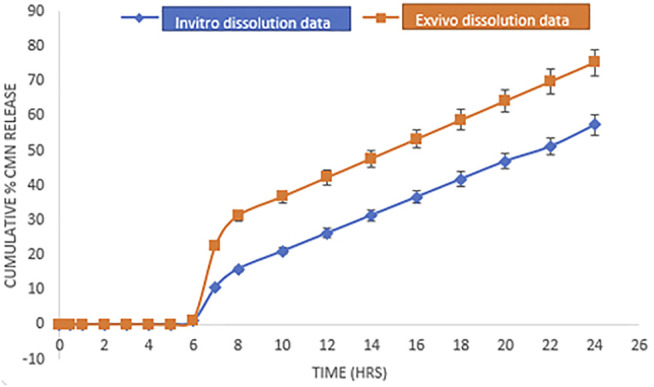
Comparative dissolution profile of M14 formulation in simulated GI fluid pH buffer media (*in vitro* dissolution data/drug release) and media containing 2% rat colon contents (*ex vivo* dissolution data/drug release). Error bars represent the standard deviation of n = 3.

The *in vitro* drug release study of the optimized CMN mucoadhesive tablets (M-14) was performed for 12 h in alkaline media (7.4 pH simulated phosphate buffer) to assess the drug release profile and its mechanism. As shown in Figure 6, at the end of 12 h, optimized M14 mucoadhesive formulations showed a sustained drug release (% CDR at 12 h 54.35%), which could be attributed to the increased hydration, expanded three-dimensional networks, and porosity of the mucoadhesive polymer blend present in mucoadhesive tablets (Kurra et al., 2019). The formation of the gel layer was observed on the surface of the formulations, which led to a longer diffusion path and a lower diffusion coefficient. Variation in the ratio of the mucoadhesive gums can cause variation in the interpenetrating networks, which in turn produces a change in the swelling properties and thus results in altered drug release (Rojewska et al., 2017; Jana and Jana, 2020).

The mechanism of drug release was explored by subjecting the data to kinetic analysis by fitting to various mathematical equations and models, viz., zero-order, first-order, Higuchi, and Peppas models. Based on higher regression values obtained, the M14 formulation followed the Korsmeyer-Peppas model (*R* ^2^ = 0.9923) of drug release with a release exponent value “n” of 0.7492, indicating that the drug release data follow the non-Fickian diffusion mechanism of drug release with anomalous diffusion type (Manwar et al., 2016). In this context, the non-Fickian diffusion through the interpenetrating three-dimensional network formed by JFM and OKM chain relaxation and increased hydration was contemplated to be the prime mechanisms governing the drug release from the formulated mucoadhesive tablets (Bartkowiak et al., 2018).

### *Ex Vivo* Outcome

The results of the study as shown in Figure 6 revealed that in both the cases, that is, the drug release study with and without 2% colonic content, CMN release was found to be less than 1% in the upper GI tract due to the pH-dependent differential swelling of mucoadhesive polymers present on the dosage form. Furthermore, after 24 h, the release of M-14 formulation in the media containing rat cecal contents was found to be 75.35%, whereas the release rate in simulated GI buffer media was found to be 57.35%. The obtained results indicate that the media containing rat cecal contents have a higher dissolution potential than that of the pH buffer media, which may be attributed to the enzymatic activity of the microflora present in the colonic contents on the mucoadhesive polymers used in the formulation (Jose et al., 2010).

The results of kinetic modeling revealed that both the dissolution profiles followed zero-order drug release (*R* ^2^= 0.951) and were fitted to the Koresemeyer–Peppas model (*R* ^2^ = 0.913). As the formulation contains swellable polymers, the n value of both the dissolution profiles appears to fall below 0.45, indicating the Fickian diffusion mechanism. A similarity factor was estimated for both the dissolution profiles by comparing them with the theoretical dissolution profile of CMN. The dissolution profile of CMN with colon contents was found to have a similarity factor f_2_ value of 92.54, indicating that the dissolution method is the best way to mimic *in vivo* colon conditions for drug release. Various microflora predominant in cecal contents, viz., *Enterobacteria* species, *Lactobacillus* species, and *Bifidobacterium* species tend to cause degradation of natural polysaccharide material, that is, JFM and OKM, resulting in effective and accelerated drug release.

### Hemoaffinity Studies

**FIGURE 7:**
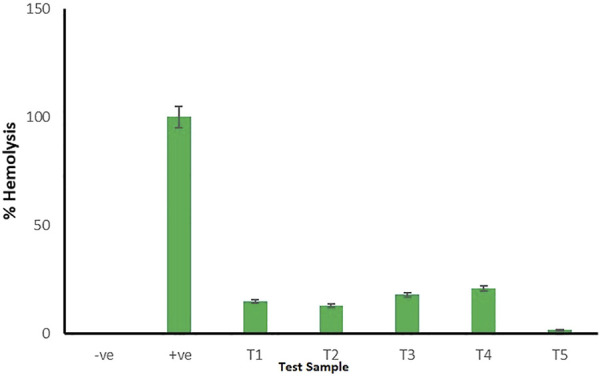
Hemolytic assay for negative control, positive control, and test samples (T1–T5). Error bars represent the standard deviation of *n* = 3.

The bioaffinity of CMN-loaded mucoadhesive tablets could be confirmed through *in vitro* hemolysis assay by evaluating the biosafety of test samples on erythrocytes. The CMN-loaded mucoadhesive tablets were observed to have high hemo-affinity with effects similar to those of the PBS, whereas the RBCs treated with double-distilled water (positive control) displayed obvious hemolysis. As shown in Figure 7, the percentage of hemolysis observed for test samples [JFM (T1), OKM (T2), JFM and OKM (T3), CMN (T4), and M14 formulation (T5)] at 541 nm was less than 5%, which falls well under the standard acceptable limit, thus suggesting its exceptional hemocompatibility. Maximum hemolysis of 3.23 ± 0.3% was observed for CMN-loaded mucoadhesive tablets. The obtained results suggest that CMN-loaded mucoadhesive tablets are suitable and safe for a wide spectrum of therapeutic applications (Dhana Lekshmi et al., 2011).

### Accelerated Stability Testing

**FIGURE 8:**
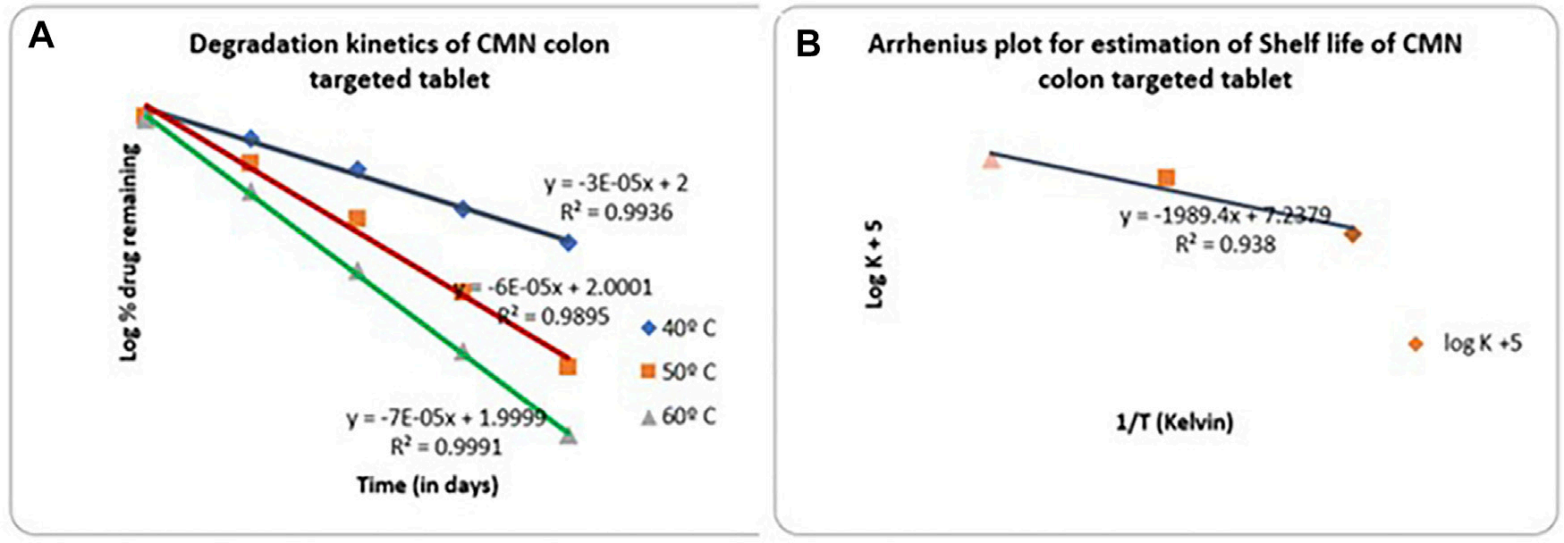
**(A)** First-order degradation kinetics plot for CMN mucoadhesive tablet formulation (M14) **(B)** Arrhenius plot for CMN colon-targeted tablet (M14).

The study was conducted to determine the shelf life of drugs in optimized CMN-layered tablets at various accelerated temperatures such as 40, 50, and 60°C. The tablets were wrapped in aluminum foil and kept in HDPE bottles, and the bottles were loaded into Remi hot air ovens. Samples were regularly withdrawn and analyzed for drug content using the UV method. The obtained stability data are shown in Supplementary Table S2. A graph is plotted by taking the log % drug remaining on the *Y*-axis and the time in days on the *X*-axis in Figure 8A. From the graph, it is observed that the drugs follow first-order degradation. The first-order degradation rate constant estimated at each temperature for CMN is given in Supplementary Table S3. From the rate constants at various temperatures, the rate constant at 25°C is estimated using the Arrhenius plot by extrapolating the line. Figure 8B gives Arrhenius plots for CMN (Higuchi and Connors, 1965). From the graphs, the rate constant at 25°C is estimated, and the shelf life of the drug is calculated from the formula. From the accelerated stability testing, the shelf life of mucoadhesive tablets of CMN was found to be 1717.67 days or 4.706 years.

### *In Vivo* Colon Targeting

**FIGURE 9:**
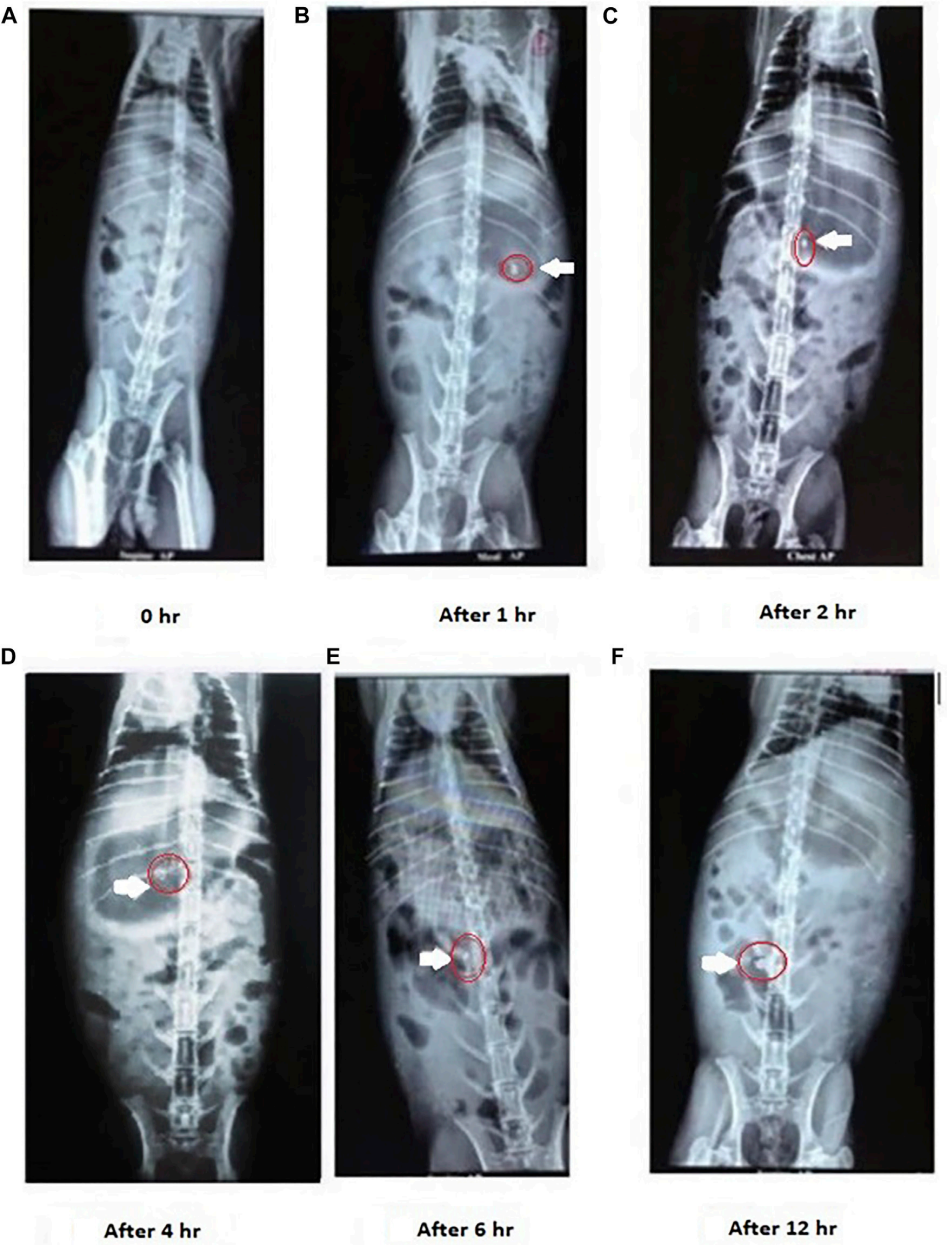
X-ray images of CMN colon targeting studies after administration of M14 mucoadhesive tablet formulation. The tablet remained intact in the gastric and upper intestinal region, On the other hand swelling and distortion in the shape of the tablet were observed in the colonic region.

The transit behavioral ability of the optimized mucoadhesive (layered) tablet of CMN, F14 was investigated in New Zealand white rabbits using an X-ray imaging technique Figure 9. The F14 formulation was reformulated by replacing CMN with barium sulfate in the mucoadhesive tablet, keeping the remaining formulation intact (Pifferi and Restani, 2003). The transit time and duration of the tablet in various regions of GIT were observed from the obtained fluoroscopic images and have been displayed in Figure 9. The tablet remained intact in the gastric and upper intestinal regions for about 3–6 h, where acidic pH was present, indicating the adhesion efficiency of the mucoadhesive tablet, and thereafter reached the colon after the intended period. Six hours post administration, it was found that the dosage form reached the colonic region. Swelling and distortion in the shape of the tablet were also observed in the colonic region, indicating the drug release in the colon region, and the same observation in similar studies has been observed (Singh et al., 2020, 2021). The X-ray images in the present study also clearly indicate that the optimized formulation was successfully targeted to the colonic region without any prior drug release.

## Conclusion

The current study is aimed to prepare and evaluate a CMN-loaded bio carrier (mucoadhesive tablet) for colon targeting using a combination of natural mucilages JFM and OKM. CSD prepared with PVP K30 enhanced the aqueous solubility of CMN present in the formulation. The Central Composite Design (CCD) and RSM were used to optimize the ratio of JFM: OKM in the mucoadhesive tablets based on the % CDR and MA as response factors. Optimized Mucoadhesive tablet (M14) exhibiting a CDR of 54.35% and an MA of 34.17 g followed anomalous non-Fickian diffusion mechanism. Comparative dissolution of the optimized mucoadhesive tablet with and without 2% colonic contents was carried out, and the results manifested a slight increase in the drug release in the presence of 2% colonic contents due to enzymatic degradation. Accelerated stability testing ensured the stability of the optimized formulation for 1717.67 days. *In vivo* drug targeting studies confirmed that the optimized formulation effectively reached the colonic region. Therefore, this study confirms the exploitation of a combination of JFM and OKM for its assuring application in the development of a colon-targeting system for macromolecules.

## Data Availability

The original contributions presented in the study are included in the article/Supplementary Material; further inquiries can be directed to the corresponding author.
