# Supplementary material for: Studies on Jackfruit–Okra Mucilage-Based Curcumin Mucoadhesive Tablet for Colon Targeted Delivery
Source: Front Pharmacol. 2022 Jul 1;13:902207. doi: 10.3389/fphar.2022.902207 (PMC9284007; doi:10.3389/fphar.2022.902207)
Supplement: Supplementary file 1 [file DataSheet1.docx]

**Appendix-A**

**Supplementary material**

***Ex vivo* dissolution studies**

Five healthy male Wistar rats weighing 150-200 g were selected for the present ex vivo study. The animals were allowed to acclimatize to the experimental environment and were provided with normal diet for few days. From the day of experimentation, the rats were fed orally with 1 ml of 2% dispersion of JFM and OKM in water. The single dose treatment was continued for duration of 7 days such that the enzymes that are specific for JFM and OKM were released. 30 min prior to the 5^th^ hour of dissolution, the rats were anesthetized and sacrificed. The abdomen of the rats was opened; colonic region was identified, ligated at both ends, dissected carefully. The cecal bag was suspended into phosphate buffered saline (PBS) which was aerated continuously. The cecal bag was opened and all the colonic contents were collected, pooled up and transferred into simulated intestinal fluid media of pH 7.4 to attain a desired dilution of 2% w/v as shown in **Figure 2**. This simulated colonic fluid is used as dissolution media. The samples were collected at regular intervals, filtered, necessary dilutions were made if necessary and were analyzed at 421 nm using UV Visible Spectrophotometry.

**Accelerated stability testing**

Stability studies were conducted for the accelerated stability testing of optimized CMN mucoadhesive tablet formulation (M-14) for colon targeting was carried out based on Q1E of ICH guidelines. Remi hot air oven was used for the purpose. The study was carried out considering accelerated temperature for a period of 60 days. Sufficient samples of formulation were prepared by packing in aluminum foil and stored in HDPE bottles at temperature of 40± 0.5°C, 50± 0.5°C and 60± 0.5°C.Samples were withdrawn at various intervals (15, 30, 45 and 60) days and analyzed for drug content by UV method. The shelf life of colon targeting CMN tablets was determined on the basis of first order degradation kinetics.

First order degradation equation: log C = Log Co - Kt/2.303 ----(2).

**Results**

**Table S1** ANOVA for response parameter % CDR (Quadratic) & MA (Linear

| **Source** | **Sum of Squares** | **df** | **Mean Square** | **F-value** | **p-value** |  |
| --- | --- | --- | --- | --- | --- | --- |
|  |  |  |  |  |  |  |
|  |  |  |  |  |  |  |
| **Model** | 1985.75 | 5 | 397.15 | 88.30 | < 0.0001 | significant |
| **A-JFM** | 1386.69 | 1 | 1386.69 | 308.31 | < 0.0001 |  |
| **B-OKM** | 366.02 | 1 | 366.02 | 81.38 | < 0.0001 |  |
| **Residual** | 12.25 | 1 | 12.25 |  |  |  |
| **Lack of Fit** | 14.402 | 1 | 1.44 | 3.2 | 0.581 | non significant |
| **Pure Error** | 5.71 | 1 | 1.71 |  |  |  |
| **Cor Total** | 2017.23 | 12 |  |  |  |  |
| **Mucoadhesive strength (Linear)** | | | | | | |
| **Model** | 437.92 | 2 | 218.96 | 136.15 | < 0.0001 | significant |
| **A-JFM** | 365.06 | 1 | 365.06 | 226.99 | < 0.0001 |  |
| **B-OKM** | 72.86 | 1 | 72.86 | 45.30 | < 0.0001 |  |
| **Residual** | 16.08 | 10 | 1.61 |  |  |  |
| **Lack of Fit** | 10.88 | 6 | 1.81 | 1.40 | 0.3896 | not significant |
| **Pure Error** | 5.20 | 4 | 1.30 |  |  |  |
| **Cor Total** | 454.00 | 12 |  |  |  |  |

**Table S2** Accelerated stability study data of CMN colon targeted tablet (M1)

| **Duration** | **40ºC** | | | **50º C** | | | **60º C** | | |
| --- | --- | --- | --- | --- | --- | --- | --- | --- | --- |
|  | **Drug Content** | **% drug remaining** | **Log % drug remaining** | **Drug content** | **% drug remaining** | **Log % drug remaining** | **Drug content** | **% drug remaining** | **Log % drug remaining** |
| Initial | 99.99 | 99.99 | 1.99996 | 99.98 | 99.98 | 1.999913 | 99.97 | 99.97 | 1.9998697 |
| 15 days | 99.91 | 99.91 | 1.99961 | 99.83 | 99.83 | 1.999261 | 99.74 | 99.74 | 1.9988694 |
| 30 days | 99.81 | 99.81 | 1.99917 | 99.65 | 99.65 | 1.998477 | 99.48 | 99.48 | 1.9977358 |
| 45 days | 99.68 | 99.68 | 1.99861 | 99.41 | 99.41 | 1.997430 | 99.22 | 99.22 | 1.9965992 |
| 60 days | 99.57 | 99.57 | 1.99813 | 99.17 | 99.17 | 1.996380 | 98.95 | 98.95 | 1.9954158 |

**Table S3** Arrhenius data for CMN Colon Targeted Tablet (M1)

| **Temperature** | **Slope X 10^-5^** | **K (day^-1^) X 10^-5^** | **Log K + 5** | **Absolute Temperature T (Kelvins)** | **(1/T) X 10^-3^** | **CMN Shelf Life at 25^o^ C** |
| --- | --- | --- | --- | --- | --- | --- |
| **40 ^0^C** | -3.10667 | 7.15465 | 0.854589 | 313 | 3.195 | **1717.67 days** |
| **50 ^0^C** | -5.93133 | 13.6599 | 1.135446 | 323 | 3.096 |  |
| **60 ^0^C** | -7.452 | 17.162 | 1.234567 | 333 | 3.356 |  |
| **Extrapolated Value at 25 ^0^C** |  | 4.8769 | 0.688143 | 298 | 3.356 |  |


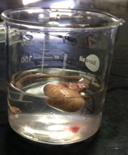

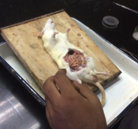

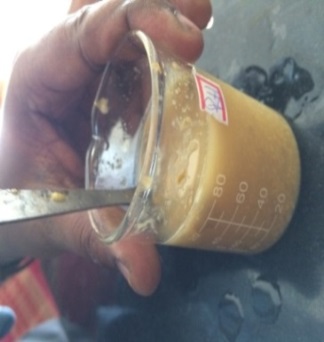


**Dissected Sprague dawley rat**

**Separated colon along with contents**

**Colonic contents dissolved in simulated dissolution medium**

**Figure S1:** Step by step procedure for preparation of simulated dissolution media with 2 % colonic contents
